# Solar cell cracks within a photovoltaic module: Characterization by AC impedance spectroscopy

**DOI:** 10.1371/journal.pone.0277768

**Published:** 2022-11-17

**Authors:** Tadanori Tanahashi, Shu-Tsung Hsu

**Affiliations:** 1 Renewable Energy Research Center, National Institute of Advanced Industrial Science and Technology (AIST), Koriyama, Fukushima, Japan; 2 Center for Measurement Standards (CMS), Industrial Technology Research Institute (ITRI), Hsinchu, Taiwan, R.O.C; Mohanlal Sukhadia University, INDIA

## Abstract

Various cell crack modes (with or without electrically inactive cell areas) can be induced in crystalline silicon photovoltaic (PV) cells within a PV module through natural thermomechanical stressors such as strong winds, heavy snow, and large hailstones. Although degradation in the performance of PV modules by cell cracks has been reported occasionally, the mode-dependent evolutions in the electrical signatures of cracks have not yet been elucidated. In this study, we propose that the reduction of the time constant in the AC impedance spectra, which is caused by the elevation of minority-carrier recombination in the p–n junction of a PV cell, is a ubiquitous signature of cracked PV cells encapsulated in a commercially available PV module. Several other characteristics derived from the illuminated current-voltage (*I–V*) and dark *I–V* data significantly evolved only in PV cells with inactive cell areas. We also propose that the evaluation by carrier recombination is a crucial diagnostic technique for detecting all crack modes, including microcracks, in wafer-based PV modules.

## 1. Introduction

As of the end of 2021, the global photovoltaic (PV) market had grown by over 940 GW owing to the annual installation of over 100 GW for the fifth successive year [1]. Further expansion of the PV market will be facilitated by advances in PV module technologies, including the development and implementation of innovative designs and materials. Within the bills of materials of the PV module, a decrease in the crystalline silicon (c-Si) wafer thickness could be a key factor leading to cost savings through the efficient use of silicon. Presently, it has reached approximately 170 μm, and a minimum thickness of 125 μm is projected to be achieved in 2032 [2]. However, the tendency to decrease the wafer thickness of PV cells could lead to a worst-case scenario with a significant reduction in the PV module/system performance owing to the initiation/propagation of cell cracks from extreme weather events (e.g., strong winds from tropical cyclones, heavily accumulated snow, and frequent hailstorms). In fact, over 20% power loss caused by cell cracks (including those attributed to hailstorms) has been occasionally reported in PV modules deployed outdoors [3–6]. Therefore, we determined the proper inspection principles for cell cracks to contribute to proactive measures in the design and manufacturing processes of PV cells/modules, as well as the appropriate operation and maintenance of PV systems.

Various detection and inspection methods for cell cracks have been proposed based on optical/imaging technologies [7–14] and electroluminescence (EL) has been the gold standard for crack detection [15]. Recently, photoluminescence (PL) and ultraviolet fluorescence (UVF) methods have been applied for the explicit identification of cell cracks in field-aged PV modules [16–18]. These techniques can provide superior qualitative information; however, quantitative data associated with the electrical characteristics of PV cells/modules are difficult to collect. Particularly, the resolution/accuracy of signals in these images is decreased in PV modules owing to their large size and interference with the encapsulant and glass within the PV modules.

As thoroughly reviewed in [9, 10, 13], numerous electrical signatures attributed to cell cracks have been reported, such as the elevations of series resistance (*R*_s_) [19–27] and saturation current densities of the second diode (*J*_02_) in the two-diode model of the PV cell/module [28–31], reduction in the short-circuit current (*I*_sc_) [19, 22, 26], shunt resistance (*R*_sh_) [30, 32–35], and fill factor (FF) [20, 36], depending on the development of cell cracks. However, the extent of power loss in PV modules with cell cracks (particularly, with microcracks) is quite small. In one study [19], the PV module power loss did not exceed 2.5%, unless all cracks were electrically isolated. It is therefore common for the degree of evolution in the electrical signatures of PV modules with cracks to be quite small. Even when obvious cell cracks with electrical isolation were identified in a commercially available PV module, the reduction in the power generated by this PV module was less than 3% [26].

In the IEC standard [15], cell cracks are rated as follows: a) Mode A (microcracks): cracks that can be detected as line defects in EL images (no electrically inactive cell regions), b) Mode B: cracks that generate at partially disconnected region from the rest of the electrical circuit, and c) Mode C: cracks that produce regions that are essentially isolated from the electrical circuit, according to the nomenclature proposed by Köntges *et al*. [19]. In this study, for the concurrent determination of indicators of the initiation and propagation of various crack modes, we aimed to statistically identify the evolution of the respective electrical signatures in individual PV cells with two different crack behaviors (PV cells with Mode B and C cracks and those with Mode A cracks), which are in a commercially available PV module degraded by sequential mechanical loading tests. Thus, we demonstrated that the elevation of minority-carrier recombination in cracked cells could be a universal indicator for all modes of cell cracks (including Mode A cracks), as discussed in detail in Section 4. Additionally, it is suggested that only this signature is evolved even in PV cells with microcracks because those in other signatures could not be detected in the PV cells. We also suggest that the identification of this evolution by AC impedance spectroscopy is more effective than conventional current–voltage (*I–V*) analyses for these PV cells. These consequences are highly likely to be equivalent to the electrical evolutions due to cell cracks in the PV modules exposed in fields because they can be obtained in individual PV cells encapsulated in a commercially available PV module.

This article is organized as follows: In Section 2, the experimental procedures for the cell -crack formation, electrical isolation of the individual PV cells within a PV module, and characterization of these PV cells are presented. In Section 3, the electrical characteristics of the individual PV cells are summarized, including the elevation of the minority carrier recombination, which is determined by the AC impedance parameters. Additionally, we discuss not only the detection mechanisms of microcracks but also the applications to practically evaluate PV modules and systems, in Section 4.

## 2. Materials and methods

### 2.1 Cell-crack formation in a PV module with nonuniform mechanical loading

A sequential mechanical loading test was conducted on a commercially available PV module(1970 × 993 × 35 mm) assembled with 72 mono-c-Si PV cells (156 × 156 mm^2^, four busbars) to form cell cracks reflecting non-uniform wind loads during a strong typhoon, as described in our previous report [37]. The *I–V* parameters—maximum power (*P*_max_), *I*_sc_, open-circuit voltage (*V*_oc_), and FF—in the PV module before this sequential mechanical loading test were assessed as 359.8 W, 9.88 A, 45.6 V, and 76.5%, respectively. Thus, the mean *P*_max_ of the individual PV cells was 4.997 W/cell, and the mean *V*_oc_ was 0.66 V/cell. In this module, no cell cracks were detected in the EL image prior to the mechanical loading test. After the non-uniform static mechanical loads with a combination of pressure load (1 h) and suction load (1 h) were applied seven times, non-uniform dynamic mechanical loads (10 cycles/min for 6 h) were applied to the PV module. The uneven mean surface pressure pattern (MSPP) used in the non-uniform static/dynamic mechanical loading can be found in the S1 Fig. The EL image was obtained at a forward bias current of 8 A. In accordance with IEC standards [15], the EL images of the respective PV cells were evaluated by the human eye with respect to the presence and severity of cell cracks.

### 2.2 Electrical isolation of the individual PV cells within the PV module

**Fig 1:**
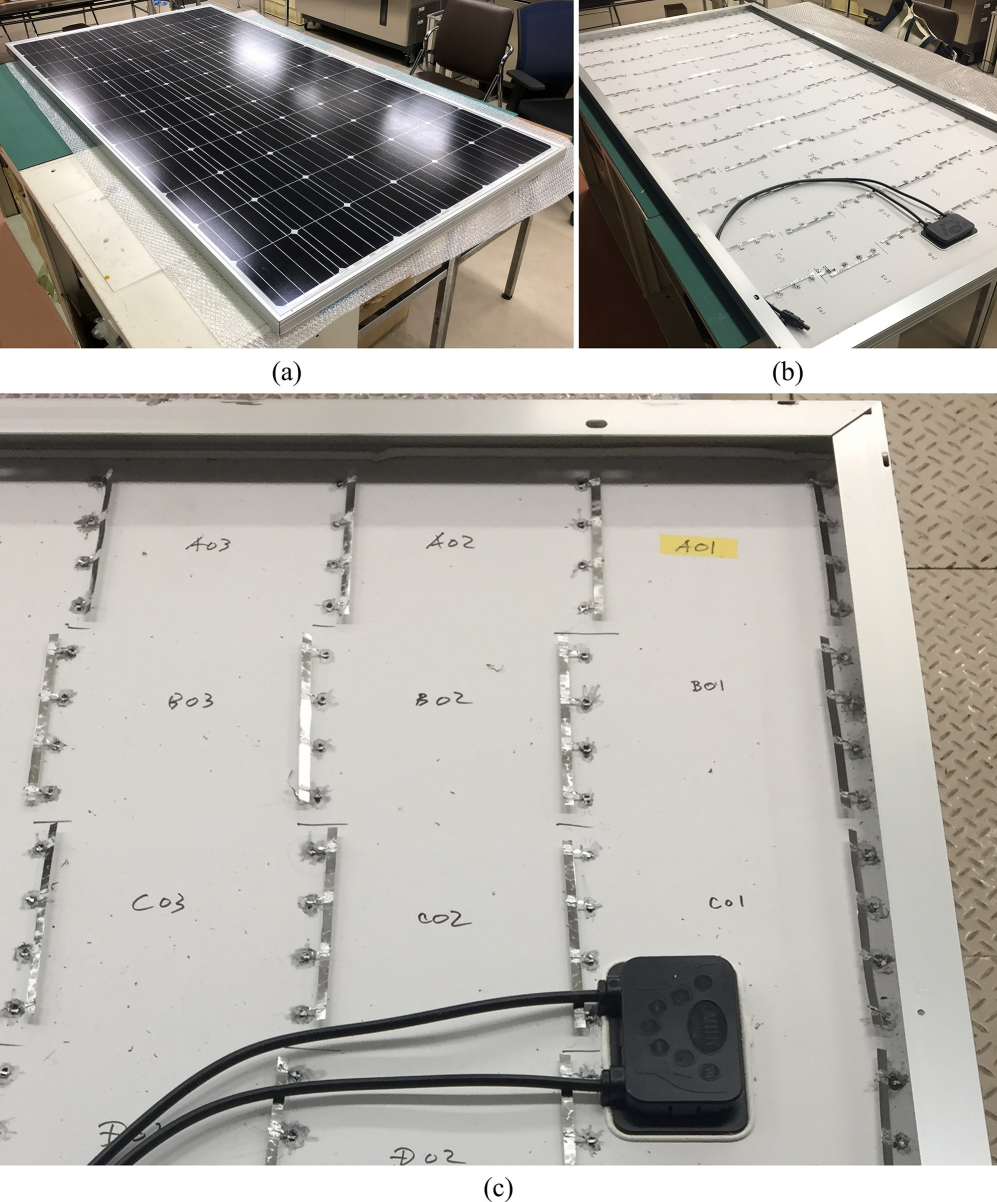
Electrical isolation of individual PV cells in a PV module with cell cracks. Front (a), rear (b), and an enlarged view of the rear (c) of the PV module are shown.

To assess the electrical characteristics of the individual PV cells within the PV module, the interconnector ribbons located in the intercell spaces of each cell string were exposed to the backsheet peeling of the corresponding portions (Fig 1), as reported in our previous article [38]. Briefly, the backsheet and encapsulant layers of the PV modules were peeled off with a micro grinder and a small wire brush to expose the interconnector ribbons between the PV cells, as can be found in S2A Fig. Copper solder leads were soldered to the exposed interconnector ribbons (S2B Fig) and connected with another set of wide copper solder ribbons at each side of the respective PV cells to evaluate the electrical characteristics of the individual PV cells, as shown in Fig 1B and 1C.

### 2.3 Electrical characterization of isolated PV cells

**Fig 2:**
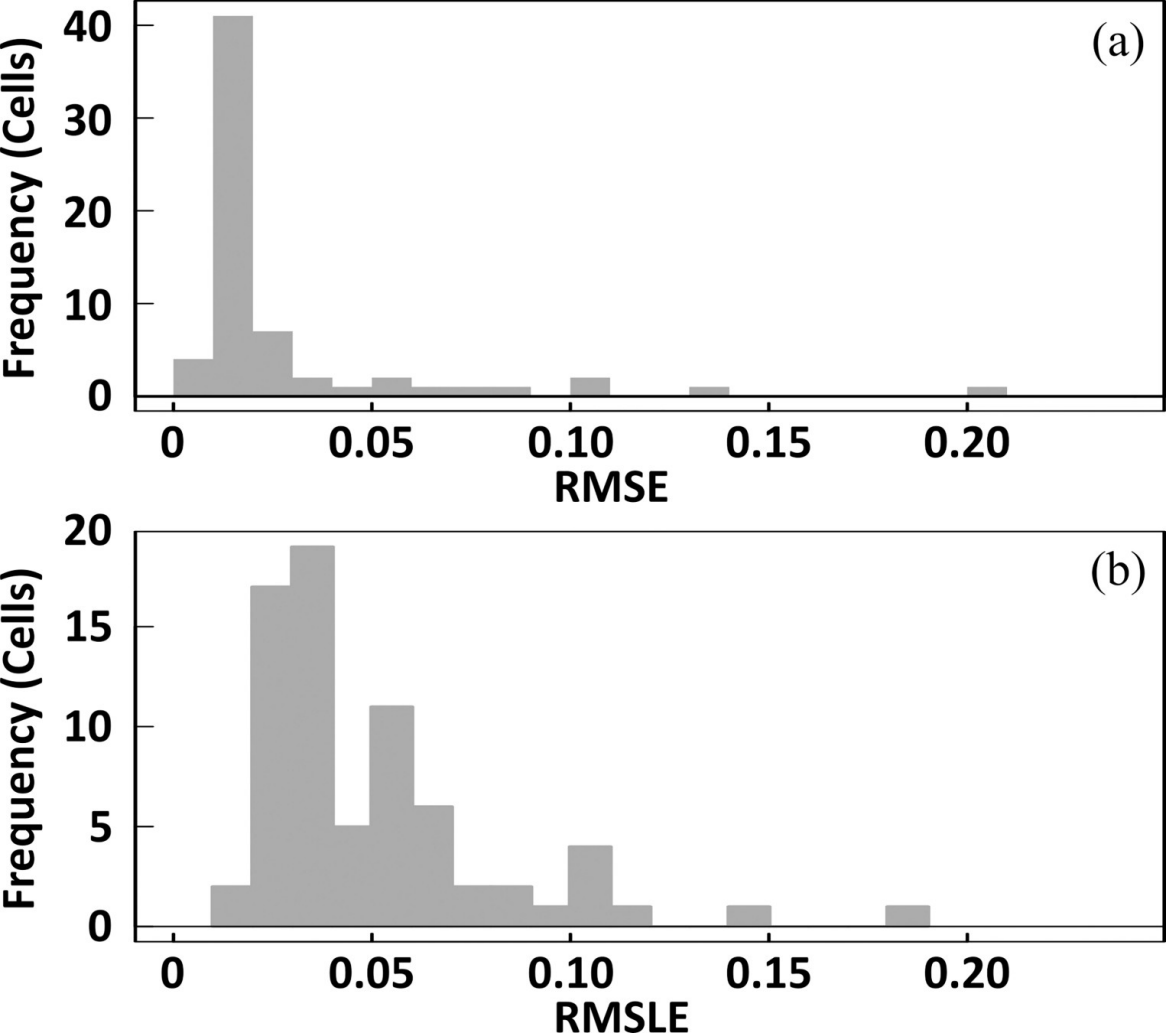
Histograms of (a) RMSE and (b) RSMLE in the illuminated and dark *I–V* curve fittings, respectively.


The *I–V* characteristics of the PV cells were individually assessed using a surface mask with an aperture area on an object PV cell, through the leads of the respective PV cells, under the standard test conditions of 1,000 W/m^2^, 25°C, and AM 1.5G [38]. Dark *I–V* data were collected at 23.3–23.9°C in the dark, by the connection of the leads of an object PV cell to a source measurement unit (Keysight B2901a). The *I–V* parameters, including *R*_s_, *R*_sh_, saturation current densities (*J*_01_ and *J*_02_), and ideality factors of diodes (*n*_1_ and *n*_2_), were extracted from the respective *I–V* data obtained under illuminated or dark conditions by fitting to two-diode equivalent circuit models as presented in (1) and (2) according to [39, 40]:

$$I_{L}=I_{ph}-\frac{V+IR_{s}}{R_{sh}}-J_{01}A\left\{exp[\frac{q(V+IR_{s})}{n_{1}k_{B}T}]-1\right\}-J_{02}A\{exp[\frac{q(V+IR_{s})}{n_{2}k_{B}T}]-1\},$$ (1)

$$I_{dark}=0-\frac{V+IR_{s}}{R_{sh}}-J_{01}A\left\{exp[\frac{q(V+IR_{s})}{n_{1}k_{B}T}]-1\right\}-J_{02}A\{exp[\frac{q(V+IR_{s})}{n_{2}k_{B}T}]-1\},$$ (2)

where *I*_L_ is the current under illumination, *I*_ph_ is the photo current, *q* is the elementary charge, *k*_B_ is Boltzmann’s constant, *T* is the temperature, *A* is the cell area, and *I*_dark_ is the current in the dark. The median root mean square error (RMSE) in the illuminated *I–V* fittings was 0.015, and that of the root mean square logarithmic error (RMSLE) in the dark *I–V* fittings was 0.040, as shown in Fig 2. These results indicate that these *I–V* parameters can be estimated with sufficient accuracy. In the dark *I–V* fitting, *n*_1_ values in the individual PV cells fell within the range of 1.18 ± 0.05, whereas the *n*_2_ values fell within the range of 1.97 ± 0.04. These values indicated that each *J*_02_ in the dark *I–V* parameters estimated from the individual PV cells corresponds to the current density, owing to the recombination current in the respective PV cells. To accurately quantify the total series resistance of each PV cell within the PV module, the lumped series resistance was calculated as *R*_s-ld_ from the respective illuminated and dark *I–V* data (3) in accordance with the procedure described by Spataru *et al*. [26]:

$$R_{s-ld}=\frac{V_{d-mp}-V_{mp}}{I_{mp}}|{}_{|I_{dark}|=I_{sc}-I_{mp}},$$ (3)

where *I*_mp_ and *V*_mp_ are the current and voltage at the maximum power point under the illuminated conditions, respectively. *V*_d-mp_ is the voltage corresponding to *I*_dark_ = *I*_sc_ − *I*_mp_ on the dark *I–V* curve.

**Fig 3:**
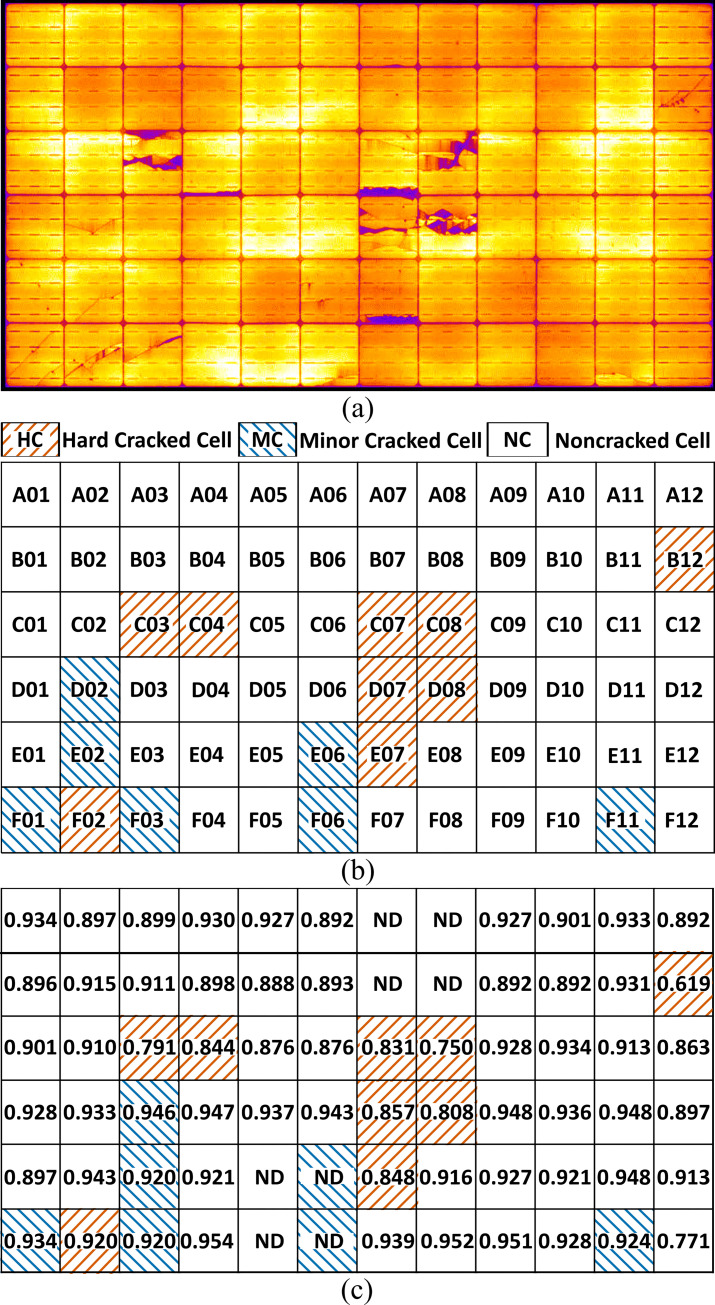
Distribution of cracked PV cells within a PV module. (a) EL image of the evaluated PV module and (b) distribution of PV cells rated with crack-behavior in the PV module. In (b), the crack-class (HC-, MC-, or NC-cell) categorized for the respective PV cells is indicated according to the legend. The label on each PV cell (A01, …, F12) denotes the respective PV cell addresses. (c) The normalized *P*_max_ for the individual PV cells is shown in each cell position within the PV module. For the PV cells labeled “ND,” *P*_max_ was not determined owing to geometrical interference by the solar simulator.

**Fig 4:**
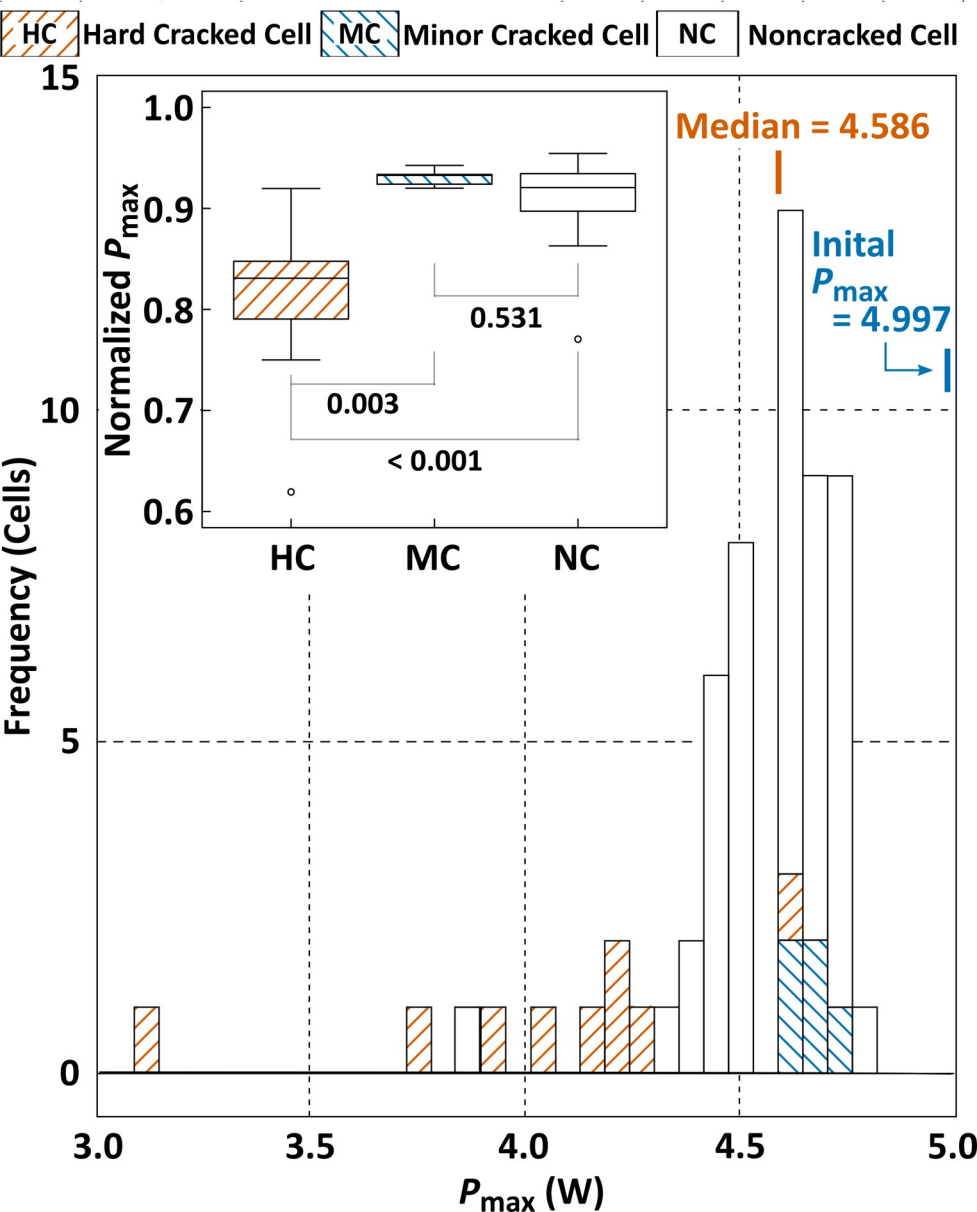
Histogram of *P*_max_ for the individual PV cells within the evaluated PV module. The crack-class (HC-, MC-, or NC-cell) rated for the respective PV cells is indicated by the top legend. The distributions of the normalized *P*_max_ are denoted as box-and-whisker plots, in a multicomparison chart for the categorized PV cells (inset). The open circles express outliers, and the figures shown in the inset chart represent the respective *p*-values in the multicomparison.

**Fig 5:**
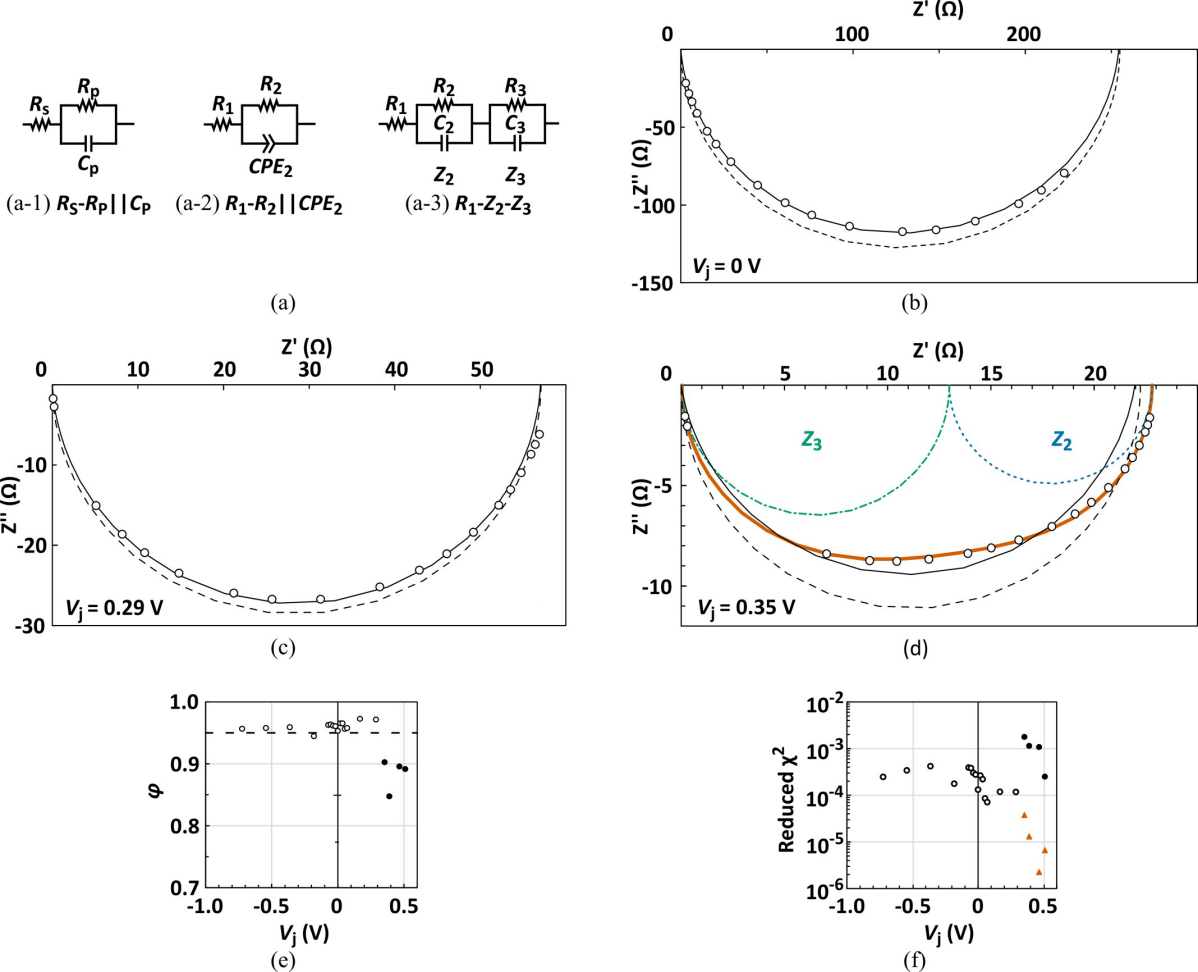
AC impedance characteristics of PV cells. (a) Postulated AC equivalent circuits for the *R*_s_-*R*_p_||*C*_p_ model (a-1), *R*_1_-*R*_2_||*CPE*_2_ model (a-2), and *R*_1_-*Z*_2_-*Z*_3_ model (a-3). (b-d) The Nyquist plots for AC impedance (open circles) measured at various forward DC bias voltages for F08 cell. The dashed and solid curves in black color indicate the model curves based on the *R*_s_-*R*_p_||*C*_p_ model (a-1) and *R*_1_-*R*_2_||*CPE*_2_ model (a-2), respectively. In (d), the blue, green, and thick-orange curves denote the model curves derived from each component (*Z*_2_, *Z*_3_) and the entire *R*_1_-*Z*_2_-*Z*_3_ model, respectively. In (e), the capacitor factor (φ) in the *R*_1_-*R*_2_||*CPE*_2_ model (a-2) for the F08 cell is indicated as a function of *V*_j_. Each reduced χ^2^ at various *V*_j_ is plotted in (f). Reduced χ^2^ from the *R*_1_-*R*_2_||*CPE*_2_ model (a-2) is shown as the open circle when *V*_j_ < 0.3 V, although those obtained at *V*_j_ ≥ 0.3 V are indicated as closed circles. Additionally, each reduced χ^2^ from the *R*_1_-*Z*_2_-*Z*_3_ model (a-3) is plotted as the orange triangle.

The AC impedance data of the individual PV cells were acquired by connecting the four-point probes of an LCR meter (Keysight 4284a with 001 DC bias option) at 22.3–24.0°C in the dark, under the condition that a 10-mV-amplitude AC voltage with various intensities of DC bias voltage was applied. According to [41], the voltage across the p–n junction (*V*_j_) of the PV cell is defined as *V*_j_ = *V*–*IR*_s_, where *V*, *I*, and *R*_*s*_ are the applied DC bias voltage, applied DC current, and series resistance, respectively (cf. *R*_s_ or *R*_1_ in Fig 5A-1–5A-3). From the AC impedance (amplitude |*Z*| and argument *θ*) collected at each frequency in the range of 20 Hz to 10 kHz, the real (*Z*’) and imaginary (*Z*”) impedances were calculated, and the AC impedance parameters in the postulated AC equivalent circuits (cf., Fig 5A-1–5A-3) were determined using a Web-based impedance-fitting application (Elchemea Analytical) [42].

### 2.4 Statistical evaluation

The Steel–Dwass test is a statistical procedure for evaluating the stochastic equality among multiple sample groups, utilizing the pairwise ranking non-parametric method. Because the distribution of the respective parameters adopted in this study is not corresponding to the normal distribution, we applied this non-parametric test to assess the difference among the distributions of these parameters, using the pSDCFlig function (Monte Carlo method with 10,000 iterations) in the NSM3 package [43] embedded into the R software [44]. Statistically significant differences were judged by the *p*-value limit (*p* < 0.05). Particularly, the multi-endpoints issue arising from post hoc definitions were not considered, since this study aimed to evaluate distinguished electrical characteristics attributed to cell cracks. The discretization of the cumulative frequency distribution to a multimodal distribution was performed using the normalmixEM function in the mixtools package [45] built into the R software.

## 3. Results

### 3.1 Power losses in the cracked PV cells

As presented in our previous report [46], various types of cell cracks were observed in the PV module after the nonuniform mechanical loading test, which included Mode B/C cracks (with inactive cell area(s) detectable in the EL image) and Mode A cracks (without any inactive cell area) [15, 19], as shown in Fig 3A. Based on the rating criteria, the individual PV cells with cell cracks were divided into two groups, particularly, the cracked cells with or without the inactive cell area were categorized as hard-cracked (HC) or minorly cracked (MC) cells, respectively. In these HC cells, the inactive areas were identified in the central region of the respective PV cells (e.g., C08 cell), as well as at the edges of the PV cells (e.g., C07 cell). The PV cells without cracks were referred to as non-cracked (NC) cells. The spatial distributions of these cell groups in the PV module are shown in Fig 3B, accompanied by the cell address, defined by the location of the respective PV cells within the PV module. Although the distribution of these cracked cells (HC and MC cells) within the PV module did not sufficiently coincide with that of the MSPP applied to the PV module, these cells are likely to be located in steep regions in the applied MSPP [46]. To quantitatively assess the extent of power loss attributed to the cell cracks, the respective maximum powers of the individual PV cells were measured and indicated in the cell matrix of the PV module (Fig 3C) as values normalized with the pristine *P*_max_ in individual PV cells (4.997 W/cell). It is recognized that obvious power loss occurred the HC cells, although that in the MC cells was not detectable at a glance, as reported in the PV module with only Mode A cracks [19].

A histogram of the actual *P*_max_ values in each PV cell is shown in Fig 4, with the mean *P*_max_ in the pristine PV cells (4.997 W/cell). A broad distribution with a long tail in the low *P*_max_ range is observed, and the median of the entire PV cell is 4.586 W/cell. Almost all HC cells were in this long tail with a low *P*_max_, although those in the MC cells were completely overlaid with those in the NC cells. This power-loss behavior was statistically confirmed by nonparametric multiple comparison analysis (see the inset figure of Fig 4); particularly, the significant decrease in *P*_max_ of HC cells from those in other cell groups was clearly demonstrated, although a substantial difference between the respective power losses in the MC cells was not proven.

### 3.2 AC impedance characteristics of the cracked PV cells

As a potential electrical signature for cell cracks, it has been reported that the intensity of the parallel resistance (*R*_p_: cf. Fig 5A-1) in the AC equivalent circuit of a PV cell/module is reduced by cell cracks that are intentionally introduced in the PV cell/module [33–35]. We then examined whether this parameter could be crucial for cell cracks (particularly, MC cells). Generally, the locus of AC impedance for a PV cell/module is expressed as a semicircle in the Nyquist plot when the AC equivalent circuit for a PV cell with a single p–n junction is assumed to be composed of a series resistance (*R*_s_) and a parallel circuit including a resistor and capacitor (Fig 5A-1: *R*_s_-*R*_p_||*C*_p_ model). Fig 5B shows the AC impedance locus observed in the PV cell without any cell cracks (F08 cell) under the conditions that the bias voltage is not applied. Although the AC impedance spectra could not be sufficiently fitted to the *R*_s_-*R*_p_||*C*_p_ model with a perfect semicircle curve (dashed curve), a satisfactory value (1.32 × 10^−4^) for the goodness of fit (also known as the reduced χ^2^) was obtained in the *R*_1_-*R*_2_||*CPE*_2_ model (Fig 5A-2), as can be observed in the depressed semicircle (solid curve) of Fig 5B. In this *R*_1_-*R*_2_||*CPE*_2_ model, because the impedance of the constant-phase element (CPE) is given by *Z*_CPE_ = 1/[*Q*(*jω*) ^*φ*^] [47], that of the *R*||*CPE* component can be described as Z_*R*||*CPE*_ = 1/[(1/*R*) + *Q*(*jω*) ^*φ*^], and the effective capacitance (C*) for the *R*||*CPE* component can be defined as C* = [(*QR*)^1/*φ*^]/*R* [48]. Where *j* and *ω* are the imaginary operator and angular frequency, respectively. Moreover, *φ* is the capacitor factor for the phase angle [*θ* = −(90 × *φ*), where *θ* is expressed in degrees unit] of the CPE impedance, with a value of 0 to 1. If *φ* = 1, the capacitance parameter (*Q*) indicates the capacitance of an ideal capacitor, that is, C* = *Q*. When *V*_j_ was applied at less than 0.3 V, a similar behavior in the AC impedance spectra was observed (Fig 5C), and *φ* estimated under these conditions was almost always over 0.95 (Fig 5E). Therefore, we deduce that the *R*_1_-*R*_2_||*CPE*_2_ model can be effectively applied into the estimation of AC impedance parameters in PV cells under these DC-bias conditions. At *V*_j_ > 0.3 V, however, we were not able to obtain a sufficient value of the reduced χ^2^ by fitting the measured AC impedance data to the *R*_1_-*R*_2_||*CPE*_2_ model (Fig 5F), and *φ* fell below 0.95 (Fig 5E). Because the effects of the low–high interface (p–p^+^ junction in the back surface field) observed in the high forward bias voltage range (at *V*_j_ > 0.3 V) should be considered, these AC impedance data were fitted to the *R*_1_-*Z*_2_-*Z*_3_ model (Fig 5A-3 and 5D), according to previous reports [49, 50]. The entire AC impedance curve (indicated by the thick curve in orange) calculated from the parameters for this model had very small values in the reduced χ^2^ (indicated by the orange triangles in Fig 5F) and was clearly divided into two curves comprising *Z*_2_ (AC impedance derived from the p−n junction) and *Z*_3_ (that from the p−p^+^ junction) in Fig 5D. Thus, to identify the critical parameters attributed to the cell cracks in individual PV cells, we used the *R*_1_-*R*_2_||*CPE*_2_ or *R*_1_-*Z*_2_-*Z*_3_ models, depending on the applied bias voltage. In all the estimated parameters of these models, the standard errors were almost always within 10%.

**Fig 6:**
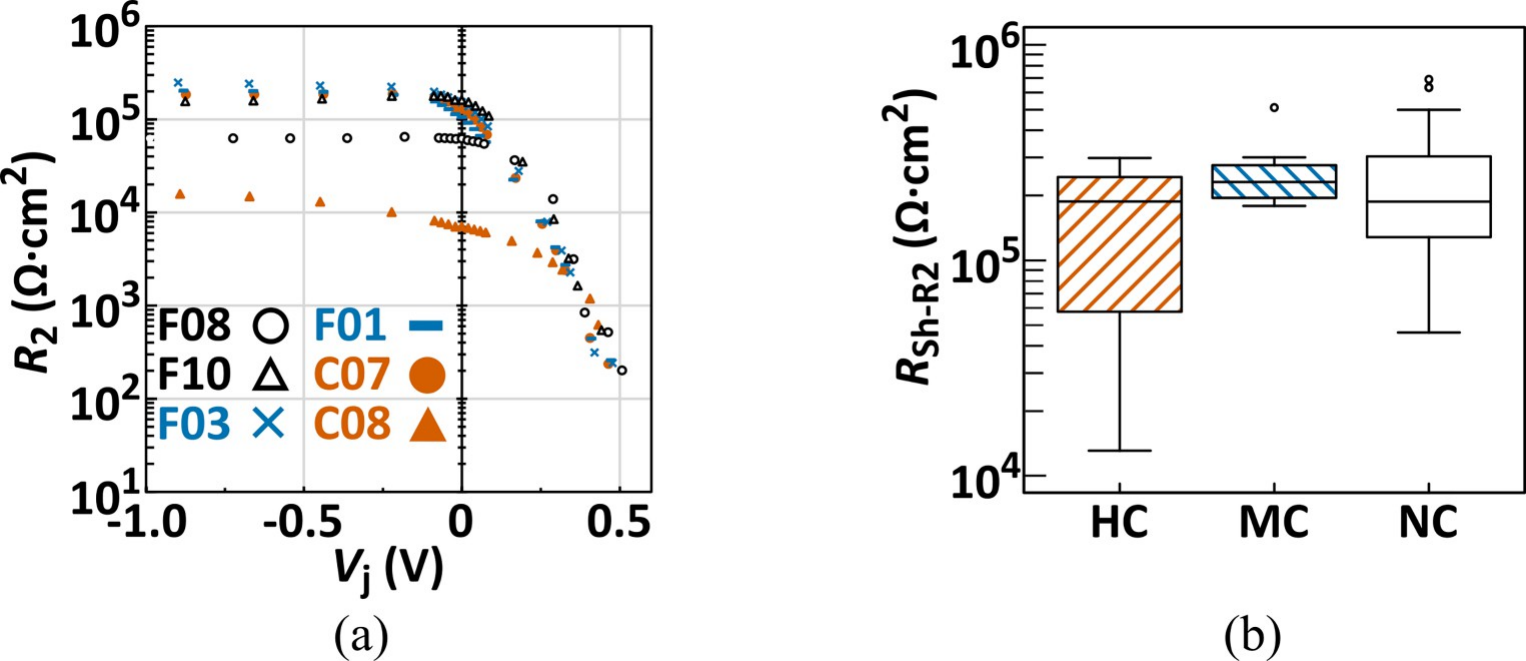
(a) Estimated *R*_2_ as a function of *V*_j_ and (b) multicomparison chart for the categorized PV cells on *R*_sh-R2_ (*R*_sh_ determined from *R*_2_). In (a), the orange, blue, and black symbols correspond to those from the two PV cells representing HC, MC, and NC cells, respectively. In (b), the open circles represent the outliers.

The dependency of the parallel resistances (*R*_2_) extracted from the AC impedance spectra of the individual PV cells on the bias voltage is shown in Fig 6A. As reported previously [51], a nearly constant resistance was estimated in the reverse bias voltage range, although drastic reductions were observed when the forward bias was applied, irrespective of the cell groups categorized as the crack mode. The extent of *R*_sh_ derived from *R*_2_ (hereafter, this *R*_sh_ is referred as *R*_sh-R2_) in the individual PV cells was numerically calculated as the mean value of *R*_2_ at −0.6. to −0.3 V in the bias voltage range [51], and their distributions in the respective PV cell categories were not significantly segregated, as shown in Fig 6B and Table 1. Although these extents are widely spread in all categories, *R*_sh−R2_ was over 1 × 10^4^ Ω·cm^2^ even in the HC cells (Fig 6B), and the *R*_sh-R2_ level was considerably higher than that proposed as the industrial *R*_sh_ criteria of the PV cell (0.8–2.4 × 10^3^ Ω·cm^2^) [30, 52–54]. Furthermore, because it is well known that the power loss attributed to potential-induced degradation (PID) with an obvious reduction in *V*_oc_ occurs when *R*_sh_ in the degraded cells/modules is below a critical value (2–3 × 10^3^ Ω·cm^2^) [55], we can conclude that the *R*_sh_ extents indicated in Fig 6B would not be a crucial cause of *V*_oc_ reduction. In previous studies [34, 35], the extent of parallel resistance (*R*_p_) was estimated from the AC impedance data collected at *V*_j_ = 0, and those in the cracked cells and modules with cracked cells were maintained at 4.86 × 10^4^ Ω·cm^2^ and ca. 6 × 10^7^ Ω·cm^2^, respectively; however these extents were decreased by approximately 40–50% of those in the pristine PV cell/module. A similar reduction in *R*_2_ measured at *V*_j_ = 0 was observed in the HC cells in our results (Fig 6A); however, a significant decrease in the MC cells was not observed compared to that in the NC cells, judging from the statistical analysis (*p* = 0.933). From these results, we conclude that the reduction in the parallel resistance (*R*_p_/*R*_2_) does not directly correlate with the occurrence of cell cracks, including microcracks.

**Table 1:** *p*-Values in multiple-comparison (Steel-Dwass test).

Parameter \ Pair	NC—HC	NC—MC	MC—HC
*P*_max_	< 0.001***	0.531	0.003**
*I*_SC_	0.039*	0.203	0.175
*I*_mp_	< 0.001***	0.304	0.018*
*V*_OC_	0.007**	0.921	0.092
*V*_mp_	< 0.001***	0.976	0.003**
FF	< 0.001***	0.817	0.004**
d-*R*_S_ ^a^	0.049*	0.765	0.404
*R*_S-ld_	< 0.001***	0.137	0.007**
d-*J*_01_ ^a^	0.953	0.986	0.977
d-*J*_02_ ^a^	0.153	0.850	0.180
d-*R*_sh_ ^a^	0.086	1.000	0.399
*R*_sh-R2_	0.495	0.514	0.401
Time Constant ^b^	< 0.001***	0.072	0.007**
Time Constant ^b^^,^ ^c^	< 0.001***		-

^a^ Prefix “d” designates the respective values determined as dark *I*-*V* characteristic.

^b^ Time constant calculated from *R*_2_ and C*/*C*_2_ at *V*_j_ = 0.4 V (cf. Fig 7).

^c^ This *p*-value is determined by the Brunner-Munzel test (on the difference in the median values) for the time constants of the cracked cells (HC- and MC-cells) and non-cracked cells (NC cells).

The number of asterisks (“*”, “**”, and “***”) represent *p* < 0.05, *p* < 0.01, and *p* < 0.001, respectively.

### 3.3 Elevation of minority-carrier recombination in cracked PV cells

**Fig 7:**
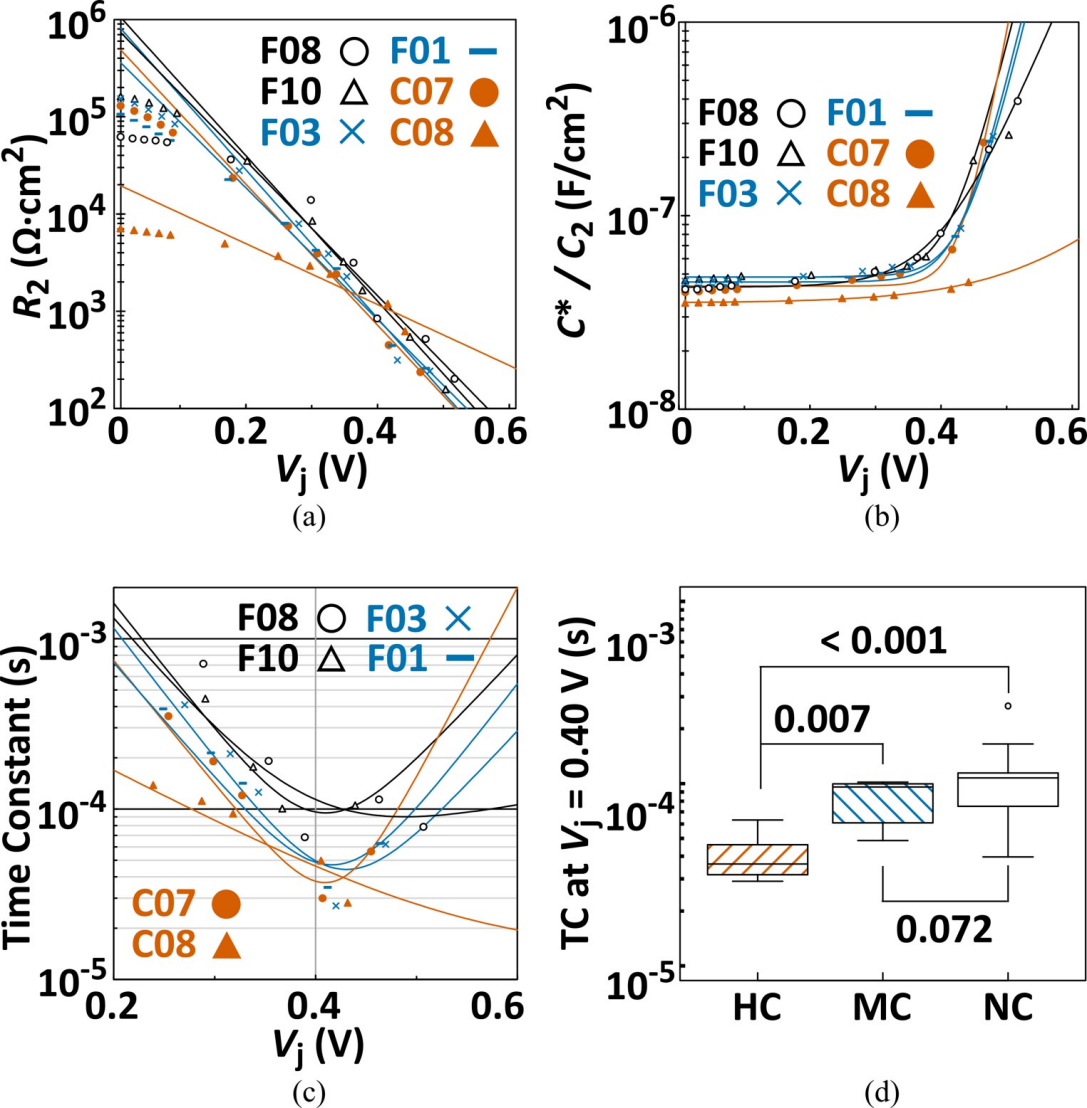
Estimated *R*_2_ (a) and C*/*C*_2_ (b) as a function of *V*_j_. Orange, blue, and black symbols correspond to those from the two PV cells representing the HC, MC, and NC cells, respectively. The time constants calculated from these *R*_2_ and C*/*C*_2_ are indicated in (c). Multicomparison charts for the categorized PV cells on the time constant observed at *V*_j_ = 0.40 V are indicated in (d). The figures in (d) denote the respective *p*-values in the multicomparison, and the open circle represents outlier.

To identify a specific electrical signature of the cracked PV cells (particularly, for the MC cells), we compared the time constants for minority-carrier recombination among these cell categories on cracks through the evaluation of the *R*_2_ and C*/*C*_2_ parameters that reflected the electrically dynamic behavior in the p–n junction of a PV cell. In the *V*_j_ range over 0.2 V, the intensity of *R*_2_ changed in a logarithmic linear manner, depending on the applied bias voltage (Fig 7A), and these data could be completely traced by the proposed relationship on the diffusion resistance in the p–n junction [41, 56]. For C*/*C*_2_, their intensities were drastically elevated in the same range as *V*_j_ (> 0.2 V), and the theoretical curve for the diffusion capacitance entirely coincided with the trends of these data [41, 56, 57], as shown in Fig 7B. For both parameters (*R*_2_ and C*/*C*_2_), we deduced that the parameters in the PV cell with complex cell cracks (in C08 and C03 cells) have a different dependency on the bias voltage from those in other PV cells. In fact, the intensities of *R*_2_ and C*/*C*_2_ were lower than those measured in other PV cells, whereas those at a higher forward bias voltage (> 0.45 V) could not be measured. This is because their comparably lower *R*_sh_ contributes to the apparent reduction of the *R*_2_ value in the forward bias voltage range, and the loading of a higher forward bias voltage (> 0.45 V) to these PV cells was difficult with the impedance spectrometer used in this study, as also suggested by Yeow *et al*. [58]. However, for the bias voltage dependencies of *R*_2_ and C*/*C*_2_ in other cells, there were small but definite differences among the crack categories. Because the time constant of the carrier recombination in the p-n junction can be simply calculated as a multiplication of the parallel resistance (*R*_2_) and capacitance (C*/*C*_2_), their bias voltage dependency in the representative cells of the respective crack categories is shown in Fig 7C. Reflecting the difference in *R*_2_ and C*/*C*_2_ among the crack categories, the time constant over the 0.3 V forward bias voltage range was distinguished between the PV cells with and without cell cracks. That is, the minimum time constants in all the cracked PV cells [including the MC cells (F03 and F01 cells)] were considerably smaller than those in the NC cells (F08 and F10 cells) at approximately 0.4 V forward bias voltage. To confirm the significant differences among these time constants observed in the respective crack categories, we statistically compared these values estimated at 0.4 V forward DC bias voltage using the nonparametric multiple comparison test (Fig 7D). A nearly significant reduction in the time constant was identified between the NC and MC cells (*p* = 0.072), which was not confirmed in other electrical signatures examined in this study, as discussed in Section 4. Simultaneously, critical reductions in the HC cells from other crack categories were also observed. These results suggest that the enhancement of the carrier recombination, which is observed through the reduction of the time constant measured by AC impedance spectroscopy, is common to cracked PV cells, regardless of the crack categories.

**Fig 8:**
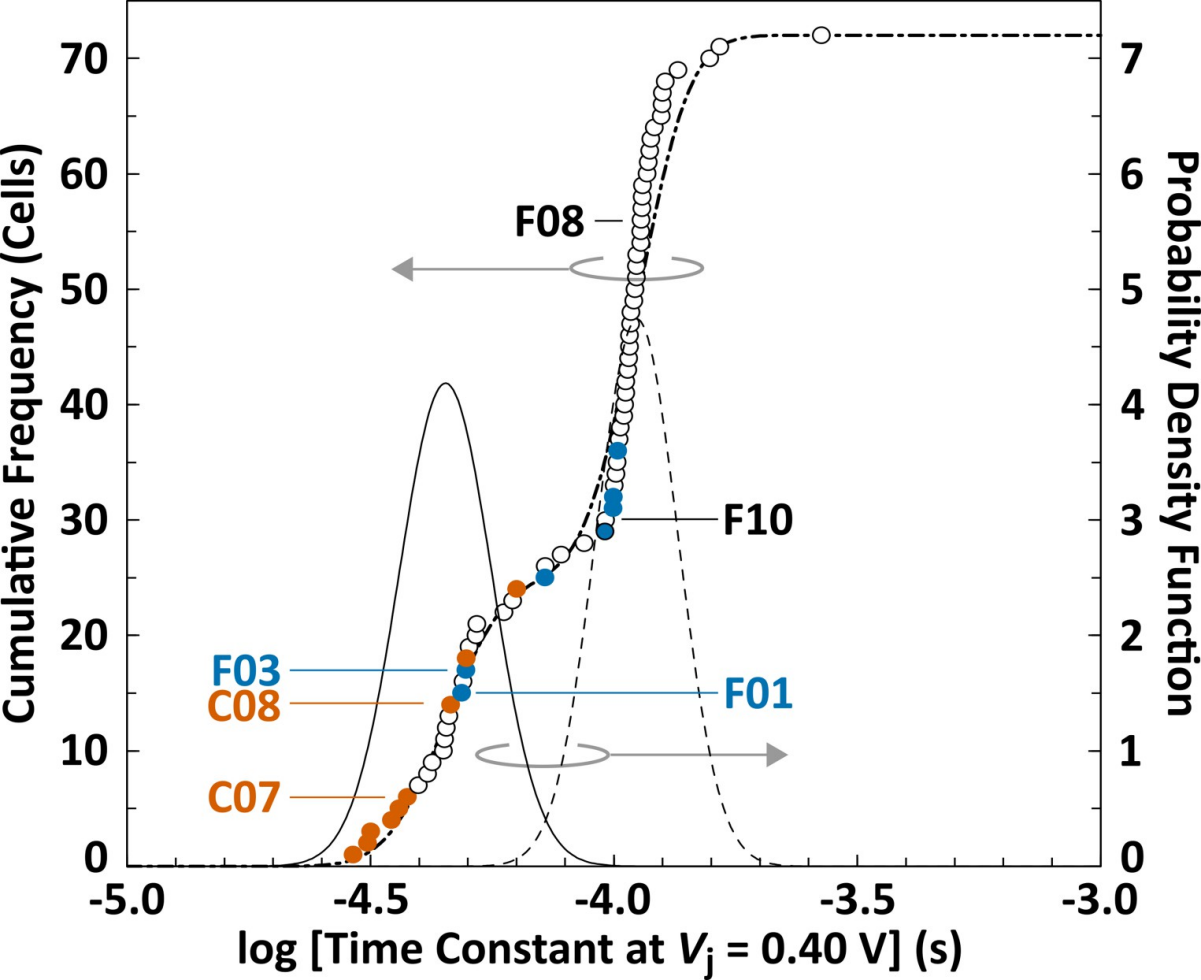
Cumulative frequency profile of the time constant determined at *V*_j_ = 0.40 V for all PV cells (left axis). Orange, blue, and open circles correspond to those in the HC, MC, and NC cells, respectively. The dashed-dotted line indicates the curve fitted to the bimodal distribution, which comprises two lognormal distributions drawn in the solid and dashed lines (right axis).

To clarify whether the reduction in the time constant of the MC cells compared to that of the NC cells is meaningful, the time constants of all the cells were plotted as a cumulative frequency profile (Fig 8). In this profile, two major breakpoints were observed at approximately 5 × 10^−5^ s (−4.3 in logarithmic scale) and 1 × 10^−4^ s (−4.0 in logarithmic scale), with a nearly flat leaning between them. This inflected profile comprises at least two distributions of the time constant. That is, there was one distribution below 1 × 10^−4^ s (lower time−constant range) and the other spread at the higher part of the time constant (higher time-constant range). This cumulative frequency profile can be almost entirely fitted to the bimodal distribution consisting of two lognormal distributions (RMSE = 3.93), as also shown in Fig 8. The parameters of the lognormal distributions are listed in Table 2. The significant difference between the population means of these distributions was confirmed by the unequal variances *t*-test (Welch’s *t*-test) with *p* < 0.001. Remarkably, the time constants of almost all cracked cells (HC- and MC-cells) located in the lower time-constant range, especially those of the HC cells were concentrated near the lower end (Fig 8). Meanwhile, a major portion of the time constants in the NC cells spread in the higher time-constant range, although some of them were in the lower time-constant range. When the statistical multiplicity arising from the post hoc analysis was considered, we confirmed that the median value (−4.308) of the time-constant distribution in the cracked cells (HC- and MC-cells) was significantly lower than that (−3.968) in the NC cells (Table 1: footnote 3) using a nonparametric statistical test (Brunner–Munzel test) for stochastic differences between two samples [59]. The mean/median values in both distributions (lognormal and real data distribution) were comparable in the respective time-constant ranges. These results suggest that the distributions located in the lower and higher time-constant ranges correspond to the electrical characteristics of the cracked (HC and MC) and non-cracked (NC) cells, respectively. Moreover, the sizes of the PV cells with cracks (including microcracks) were estimated to be 25 out of 72 (cells) when all the PV cells with cell cracks were assumed to be distributed in the lower time-constant range (Table 2). This indicates that the recall rate in our rating was 64% [= 16 cells (consisting of nine HC cells and seven MC cells) / 25 cells]. Because this rate is comparable to those (67 ± 15%) reported in a previous article [60], there is no contradiction between the cracked cell sizes estimated from the bimodal distribution of the time constants and those presumed by the actual inspection. Consequently, we deduce that the remarkable decrease in the time constant of the carrier recombination should be recognized as a specific electrical signature for all cracked PV cells (including MC cells).

**Table 2:** Parameters of two lognormal distributions.

Parameter	Time Constant Range	
	Low	High
Mean (s)	4.51 × 10^−5^	1.21 × 10^−4^
	−4.345 ^a^	−3.953 ^a^
Standard Deviation (s)	0.0953	0.0842
Estimated Size (Cells)	25	47

^a^ These values indicate the logarithm of the respective means.

### 3.4 *I*–*V* characteristics of the cracked PV cells

**Fig 9:**
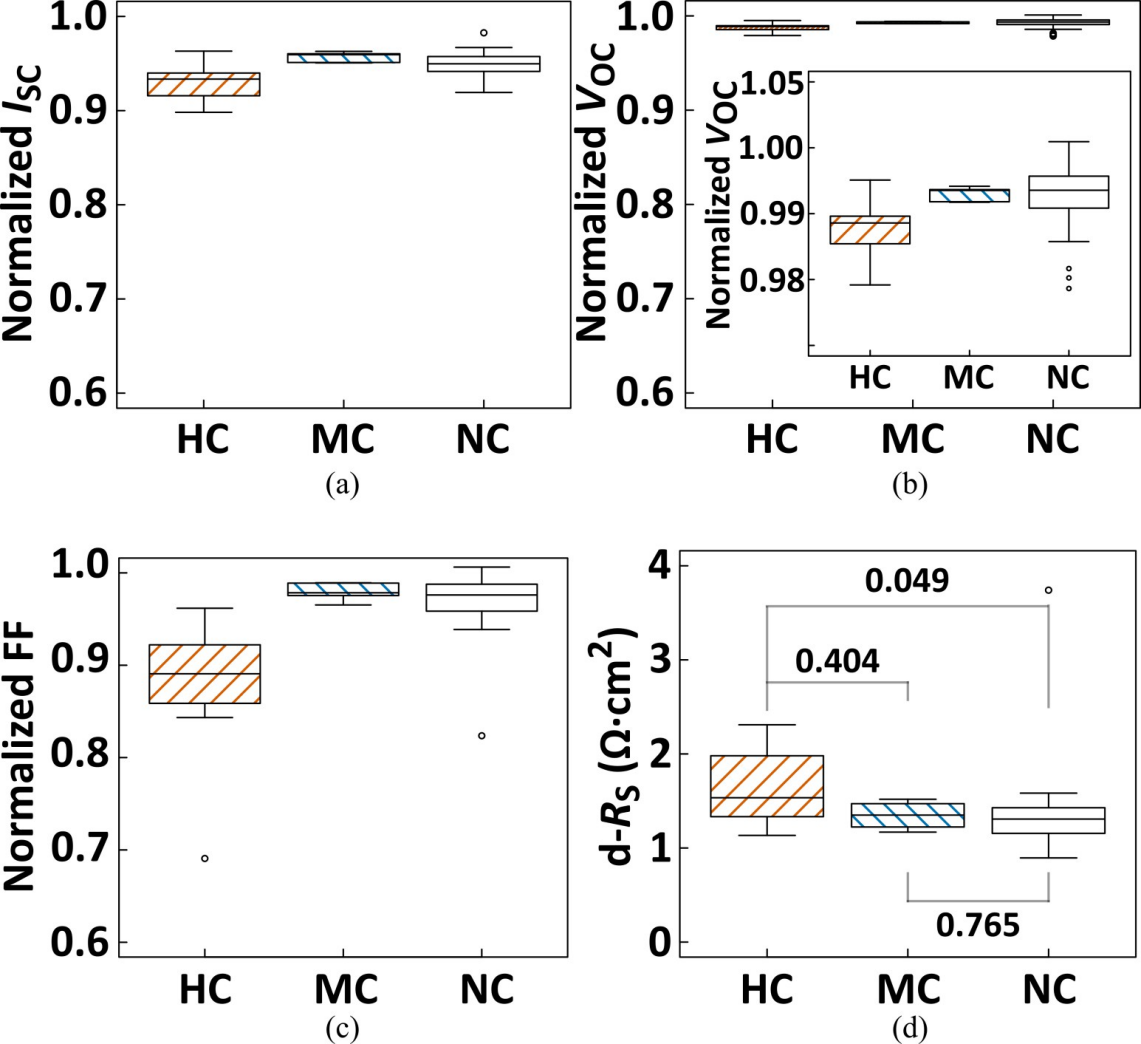
Multicomparison charts for the categorized PV cells on (a) *I*_sc_, (b) *V*_oc_, (c) FF, and (d) d-*R*_s_ (*R*_s_ determined from dark *I–V* data). Subfigure (b) with a vertically expanded axis is also shown as the inset. The open circles represent outliers, and the figures in (d) denote the respective *p*-values in the multicomparison.

The *I–V* parameters of the individual PV cells within the PV module subjected to the nonuniform mechanical loading test (Fig 3) were extracted from their *I–V* curves obtained under the illuminated and dark conditions (hereafter referred to as flash *I–V* and dark *I–V*, respectively); these are summarized in Fig 9, and all *p*-values estimated by the Steel–Dwass test are added to Table 1. As potential electrical signature for cell cracks, the critical reduction *I*_mp_ in the Mode B and C cells laminated in the mini-PV modules has been recently reported [61]; however the authors did not mention the evolution in the Mode A cells. In this study (Table 1), a significant decrease in the *I*_mp_ was confirmed in the HC cells (Mode B and C cells), although no significant difference between the MC (Mode A cells) and NC cells was verified. There was also a significant difference in the *I*_mp_ between the MC and HC cells. Del Prado Santamaria *et al*. demonstrated that the decrease in the *I*_sc_ was a specific feature in Mode C cells [61], and it was also confirmed in our study that *I*_sc_ was decreased in the HC cells (Fig 9A and Table 1), but not in the MC cells. Based on the combination of two independent observations (in [61] and this study), we infer that *I*_mp_ reduction is a common signature in HC cells, and the crucial reduction of *I*_sc_ could be attributed to the occurrence of an electrically inactive area within the HC cells. Furthermore, *I*_mp_ reduction cannot be regarded as a distinguishing feature of MC cells. Similar to the *I*_sc_ case, obvious decreases in *V*_oc_, *V*_mp_, and FF were statistically identified in their distributions in the HC and NC cells. For the MC cells, substantial reductions against the NC cells were not statistically detected. Thus, for the *I–V* parameters extracted from the flash *I–V* curves, we did not identify any common feature(s) caused by the microcracks (3rd column in Table 1).

**Fig 10:**
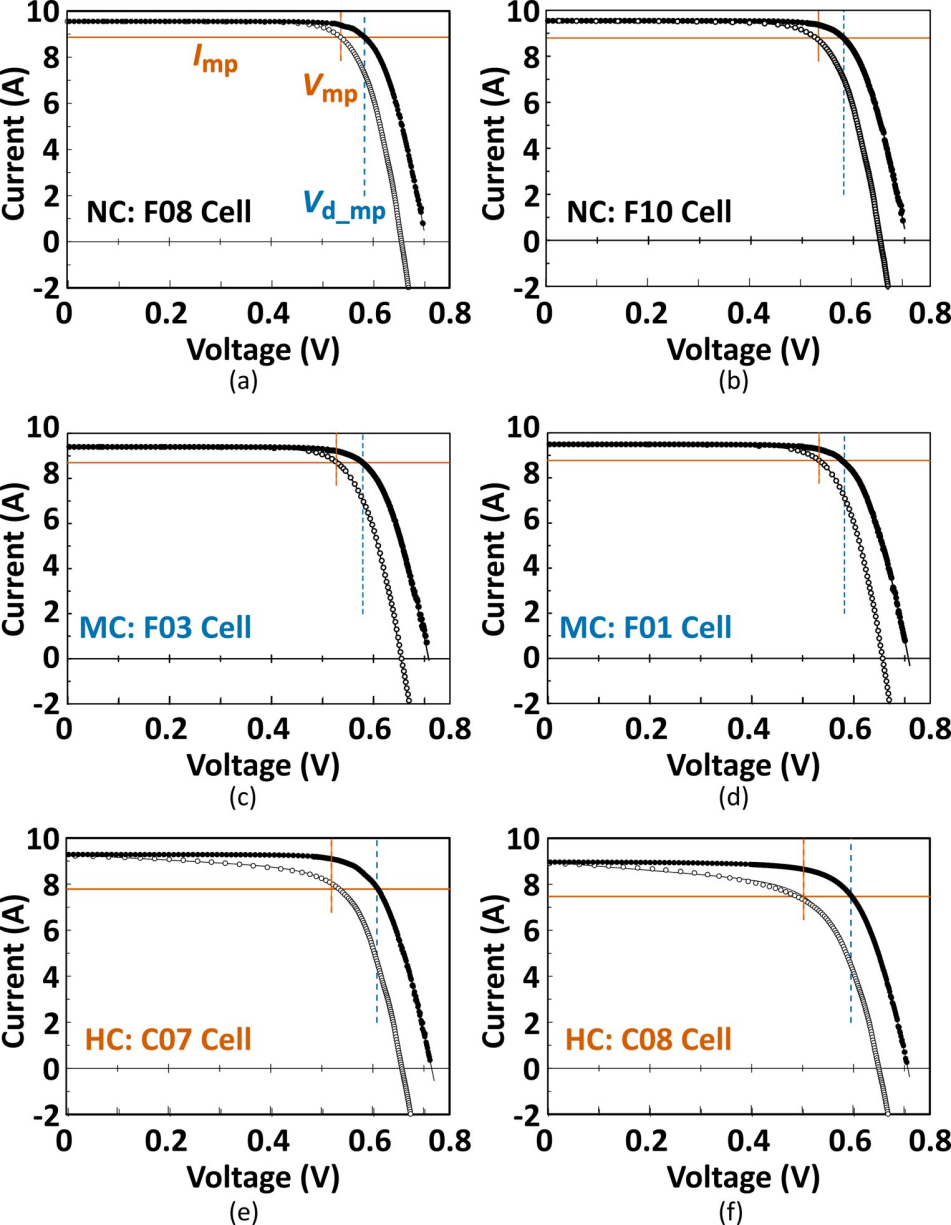
Flash *I–V* data (open circles) and overlaid “(*I*_L_-*I*_dark_) vs. *V*_dark_” data (closed circles) for two PV cells representing NC, MC, and HC cells. The corresponding fitted curves are indicated by black lines. The vertical orange solid-lines and blue dashed-lines denote *V*_mp_ and *V*_d-mp_, respectively, for each PV cell, and the horizontal orange solid-lines also represent *I*_mp_ for the respective PV cells.

**Fig 11:**
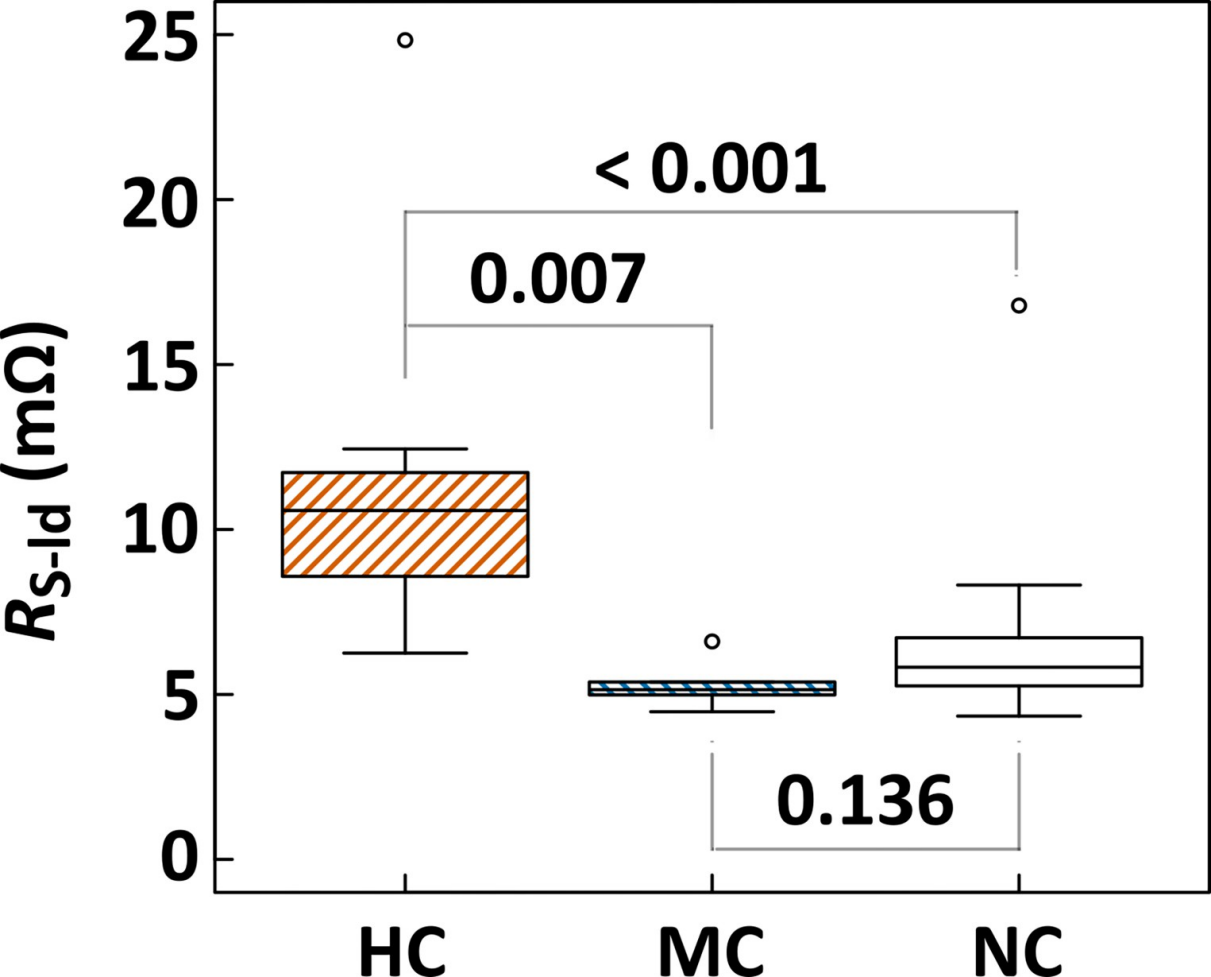
Multicomparison results for all categorized PV cells on *R*_s-ld_. Respective *p*-values are also indicated. The open circles express outliers.

Among the flash *I–V* parameters indicated in Fig 9A–9C, the most intense decrease in the HC cells was observed in the FF case. Because FF reduction depends on the evolution of *R*_s_ and/or *R*_sh_, we statistically analyzed the correlation between the crack behavior and the extent estimated from the dark *I–V* data. No significant deviation was observed among all cell groups in the *R*_sh_ case (d-*R*_sh_ in Table 1), as well as those estimated from the AC impedance data (*R*_sh-R2_ in Table 1). The significant elevation of *R*_s_ (*p* = 0.049) in the HC cells was validated, as shown in Fig 9D; however, the obvious evolution of *R*_s_ was not confirmed in the MC cells (*p* = 0.765). To critically assess the contribution of *R*_s_ elevation in the MC cells, we estimated the total *R*_s_ value (*R*_s-ld_) in these cell groups (Figs 10 and 11), which was suggested as the lumped *R*_s_ around the maximum power point [26, 62].

In Fig 10A–10F, the (*I*_L_−*I*_dark_)–*V*_dark_ curves are drawn with the flash *I–V* curves for the respective PV cells, in accordance with a previous report [26]. Here, *I*_L_, *I*_dark_, and *V*_dark_ are the maximum currents determined from *I*_sc_ in the flash *I–V* data, and the current and voltage on the dark *I–V* curve, respectively. The *R*_s-ld_ was calculated using (3). Although the values of *I*_mp_, *V*_mp_, and *V*_d-mp_ were nearly equal between the NC cells (F08 and F10 cells) and MC cells (F03 and F01 cells), *I*_mp_ and *V*_mp_ decreased in the HC cells (C07 and C08 cells). Hence, because an increase in *V*_d-mp_ was also observed in the HC cells, *R*_s-ld_ was drastically elevated only in the HC cells (Fig 11). A significant elevation in *R*_s-ld_ was not detected in the MC cells with obvious microcracks. As shown in Fig 10E and 10F, the divergence between the flash and dark *I–V* curves at the low voltage range (0 to 0.4 V) is clearly demonstrated in the HC cells with high *R*_s-ld_; this phenomenon is known as “Fake Shunt” which is caused by the spatial inhomogeneous distribution of *R*_s_ in a PV cell/module [63, 64]. Because this “fake shunt” was not recognized in the MC cells, it was confirmed that a noticeable non-uniform elevation in the *R*_s_ did not occur in the individual MC cells.

**Fig 12:**
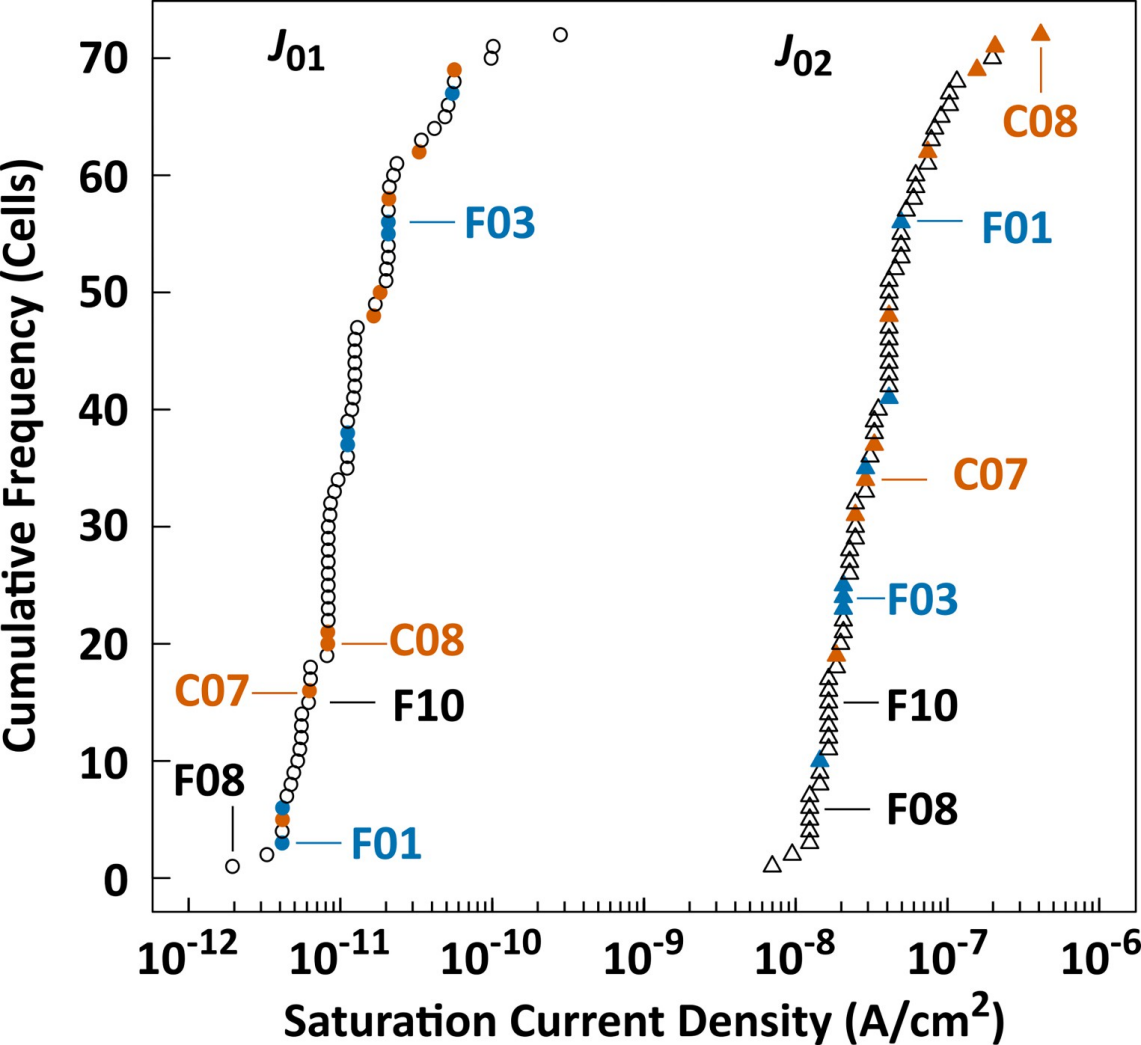
Cumulative frequency profiles of the saturation current densities [*J*_01_ (circles) and *J*_02_ (triangles)] determined from dark *I*–*V* data. Orange, blue, and open symbols correspond to those in the HC, MC, and NC cells, respectively.

The elevation of *J*_02_ was reported as a crucial electrical signature for a PV cell with microcracks, which can be observed in the global *I–V* curve, as well as in the local *I–V* image obtained in the microcrack region [29]. The values of *J*_01_ and *J*_02_ were estimated from the dark *I–V* data of the individual cells, and are indicated as cumulative frequency profiles (Fig 12). Both frequencies monotonically proliferated with an increase in the respective saturation current densities, and no obvious flection was observed. Additionally, an uneven distribution owing to the difference in the crack categories was not observed in the *J*_01_ and *J*_02_ cases. Accordingly, *J*_02_-elevation in both the HC and MC cells was not significantly identified in our data (Table 1), and *J*_01_-elevation in both cell groups was not statistically evident. In this subsection, when PV cells are encapsulated as a PV module, we demonstrate that the peculiar electrical signatures of the individual PV cells with microcracks cannot be practically identified by the electrical parameters derived from the conventional *I–V* curve analyses, but those of the PV cells with an electrically inactive area can be recognized easily.

## 4. Discussion

For PV cells encapsulated in a PV module, we demonstrated the evolution of various electrical signatures in PV cells with (MC and HC cells) or without (NC cells) cell cracks. In this study, the evolution from the pristine state of the respective PV cells has not been directly shown because we applied a typical destructive analysis (the electrical isolation of each cell from the electrical circuit of a PV module); the confounding factor(s) may affect the evolution of these signatures. However, we can presume that the evolutions identified in this study are attributed to cell cracks for following three reasons: 1) Each electrical signature of all the PV cells within a commercially available PV module can be presumed to have similar values with a certain deviation range, similar to a normal distribution, because the latest PV modules are manufactured under good quality control conditions. In fact, the saturation current densities (*J*_01_ and *J*_02_) of all PV cells have their respective monomodal distributions with a small variation range, and those of the cracked PV cells cannot be distinguished from those in the noncracked PV cells (Fig 12). 2) Although there is a bias in the distribution of the electrical signature of the respective PV cells within a pristine PV module, it is unlikely that the PV cells in the biased positions of the distribution would meaningfully correspond to the PV cells with cracks induced by mechanical stress. However, the time constant in the cracked PV cells is confined to one side of the distribution (Fig 8). 3) Significant evolutions of the electrical signatures (*P*_max_, *I*_sc_, *I*_mp_, FF, and d-*R*_s_) were observed in the cracked PV cells with electrically inactive regions, coinciding with the results reported in previous publications (in particular, in [61]). Therefore, we conclude that the evolution of these electrical characteristics, which were observed in this study, should be predominantly used to study cell cracks.

As recently reported in [60], the recall rates for crack detection by conventional ELs, PLs, and high-resolution EL were 67 ± 15%, 74 ± 18%, and 84 ± 3%, respectively, even for plain PV cells prior to encapsulation to assemble a PV module. This suggests that these methods fail to detect 16–33% of the actual cracks placed in the PV cells and that the miss rate is likely to be higher when targeting cracks in the PV cells within the PV module because of the reduced resolution of the images. Furthermore, the inspection method for cell cracks should have a broad spectrum to detect various types of crack modes because the cells with different crack modes are mixed within a PV module damaged by thermomechanical stress. Within this context, Spataru *et al*. clearly identified the distinguishing evolutions of electrical signatures in PV modules depending on the initiation/propagation of cell cracks [26]; the combined evolution (slight reductions in *I*_sc_, *V*_oc_, and FF, accompanied by drastic elevations in *R*_s-ld_ and *J*_Loss_) was a unique signature of cell-crack development. Although this criterion is valuable for determining the specific degradation modes (cell damage) from various failure modes, it has not yet been proven whether this criterion can cover all cell-crack modes, especially microcracks. In fact, although this combined evolution was clearly confirmed in PV modules with a large power loss, it can be observed that the evolution of the dark *I*-*V* curve in the PV module with a small power loss was considerably smaller [26, 65]. Moreover, it was suggested in their recent report that these evolutions have limitations when applied in cell-crack analysis [61]. This implies that microcracks could not be clearly detected by the evolutions of the dark *I*-*V* parameters (particularly, *J*_01_ and *J*_02_) when they occurred in the PV cells encapsulated in a PV module, as shown in Fig 12. In this study, because the electrical characteristics were individually assessed in the respective PV cells with various crack modes, we can conclude that the evolution of the carrier recombination is a universal electrical signature throughout all the cell-crack modes. In other words, it is suggested that microcracks can be practically detected only by this signature, but not by other electrical signatures. Furthermore, the evolution of other signatures (including the obvious decrease in output power) can be detected, depending on the cell-crack propagation (e.g., the emergence and expansion of electrically inactive cell area) due to further thermomechanical stress [31, 66].

So far, various electrical signatures attributed to cell cracks have been reported, such as a possible decrease in *I*_sc_ owing to the development of an inactive cell area [19, 22, 26], an increase in *R*_s_ [19–27], and a reduction in *R*_sh_/*R*_p_ [30, 32–35]. The *J*_02_ elevation, which reflects the increase in carrier recombination occurring at the p-n junction, has been clearly demonstrated at the PV cell level [28–31]; however, at the PV module level, it may not necessarily apply to all cell-crack modes (including microcracks) [26, 61, 65], as discussed above. At the PV cell level, it is suggested that the evolution of these signatures (excluding carrier recombination and its relevant ones) depends on the progression of the crack modes, that is, the signatures (such as *R*_s_-elevation) would appear if the cracks reached the rear side from the front surface of the PV cells [30, 31]. Our findings are consistent with this suggestion. Even in the PV cells within a PV module, a clear tendency toward elevated carrier recombination was observed in all cracked PV cells, although those in the other characteristics could not be significantly recognized in the PV cells with only microcracks. Thus, this increase in carrier recombination is caused by the newly generated recombination centers in the microcracks [28–30]. Particularly, because the occurrence of microcracks is equivalent to the generation of a new edge in the PV cell, the non-passivated surface of silicon should be exposed. An increase in carrier recombination was observed when PV cells were cut with laser scraping equipment [67, 68], and it was deduced that this elevation predominantly proceeds in the depletion layer of an edge in the PV cells [69]. Additionally, the debris accumulated within the cracks of the PV cells would also act as a recombination center [70]. Therefore, we presume that this elevation is identified in all crack modes, not just in microcracks that have not yet propagated. However, the evolution of other signatures depends on the propagation of microcracks, such as the rear-side relevant phenomena (e.g., the formation of “metal-to-metal contact” [30], the partial fracture of the Al layer coated on the rear side of the PV cells [25]), and/or the broad isolation of an electrically inactive cell area [22].

For the accurate detection of carrier recombination occurring at the depletion layer located within a PV cell, AC impedance spectroscopy should be selected from various methods, because the diffusion/transition capacitances can be satisfactorily estimated when the forward or reverse DC bias voltage is applied concurrently for the estimation of dynamic/shunt resistances [71]. Moreover, using this approach, the AC impedance parameters in the p–n junction can be independently extracted from those in the p–p^+^ junction placed at the interface between the Si bulk (p) and rear Al-BSF (p^+^) [50]. The evolution of electrical signatures caused by cell cracks has been evaluated by AC impedance spectroscopy analysis [33–35]; however, because AC impedance parameters (*C*_p_/*R*_p_/*R*_s_ in the AC equivalent circuit, cf. Fig 5A-1) were extracted from the AC impedance spectra obtained under DC biasless conditions, it has been advocated that the drastic reduction of *R*_p_ might correlate with the extent of the cracks. In this study, we applied a forward/reverse DC bias voltage at several voltage levels when the AC impedance data were acquired to estimate the AC impedance parameters in the p–n junction. Consequently, although the increase in the carrier recombination was not confirmed in the evolutions of *J*_01_ and *J*_02_ in the cracked PV cells, we statistically demonstrated that a trend toward significance in the reduction of the time constant is a critical signature of cell cracks through the estimation of their evolutions occurring in the p-n junction from those in an entire PV cell (including the p–p^+^ junction).

Because microcracks can be propagated through their penetration into the deeper bulk layer of PV cells by the thermomechanical stress experienced in laboratory tests and on-site (including transportation, installation, and/or operation stages) [10, 72], the evolution of carrier recombination, as a favorable signature of microcracks, is a crucial indicator/predictor to proactively maintain PV modules, even when the microcracks cannot be detected in their EL images. Because the data acquisition in AC impedance spectroscopy has been performed on PV modules [34, 73–75], the measurement accomplished using a DC bias voltage can be conducted effortlessly. Therefore, the diagnosis of failures with cell cracks at both the PV module and string levels, which is based on our conclusions, could be practically implemented for the assessment of the condition of a PV plant, the estimation of needed repairs, and asset transfer, although further verification in actual PV modules and strings is necessary. Moreover, this diagnostic indicator could also be a pass/fail criterion for quality assurance in processes including the manufacturing, transportation, and installation of PV modules.

## 5. Conclusions

In this study, we observed a remarkable reduction in the AC impedance spectroscopy time constant for all cell-crack modes including microcracks, although the evolutions in other electrical signatures were not meaningfully related to microcracks in the PV cells within a PV module. Because this reduction reflects the elevation of the minority-carrier recombination at the p-n junction in the c-Si PV cell, we deduced that cell cracks located in a PV cell can be quantitatively assessed using this electrically measurable signature. This work is the first attempt to comprehensively elucidate the electrical behavior of cracks located in individual PV cells encapsulated in a PV module mimicking wind-load damage, by AC impedance spectroscopy with various DC bias voltages. Therefore, the signature identified in this procedure could be a valuable indicator and beneficial technique for assessing the health of PV modules and systems through practical verification.

## Supporting information

S1 Fig MSPP (in Pa) for the pressure load (a) or the suction load (b). Alphanumeric characters outside the box indicate the PV cell address shown in Fig 3.
(DOCX)

S2 Fig Interconnector ribbons exposed using a micro-grinder (a) and the attached electrical leads (b).
(DOCX)
