# Supplementary material for: Solar cell cracks within a photovoltaic module: Characterization by AC impedance spectroscopy
Source: PLoS One. 2022 Nov 17;17(11):e0277768. doi: 10.1371/journal.pone.0277768 (PMC9671427; doi:10.1371/journal.pone.0277768)
Supplement: S1 Fig — MSPP (in Pa) for the pressure load (a) or the suction load (b). Alphanumeric characters outside the box indicate the PV cell address shown in Fig 3. (DOCX) [file pone.0277768.s001.docx]

S1 Supporting Information for

“Solar cell cracks within a photovoltaic module: Characterization by AC impedance spectroscopy”

Tadanori Tanahashi, Shu-Tsung Hsu

For the sequential mechanical loading test, the non-uniform pressure/suction load was applied to the PV module under test, by a homemade non-uniform dynamic mechanical loads system with eighteen (3 × 6) pistons having four suction cups in each piston [1], according to the MSPP indicated in Fig S1.


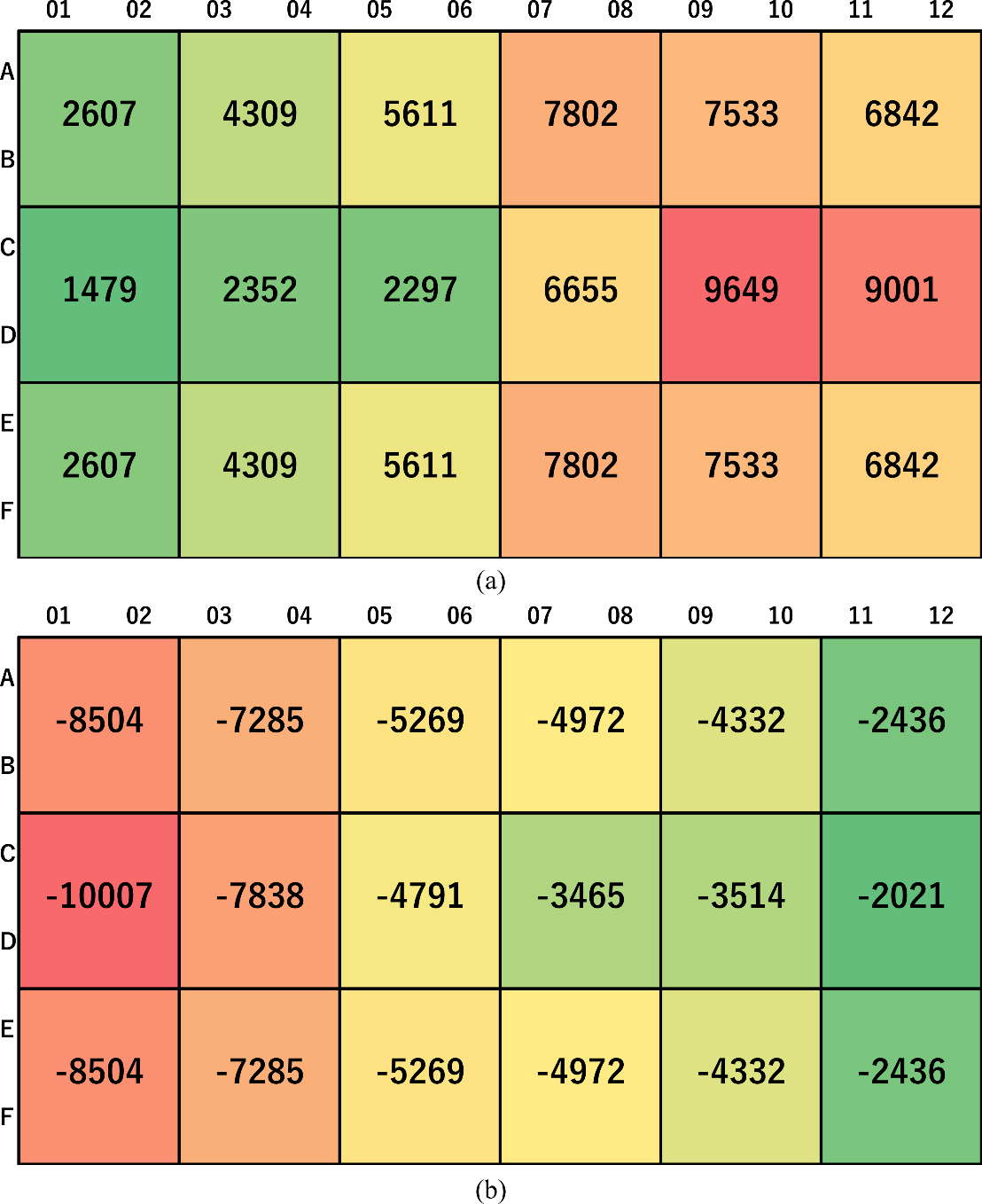


**Fig S1. MSPP (in Pa) for the pressure load (a) or the suction load (b).** Alphanumeric characters outside the box indicate the PV cell address shown in Fig 3.

1. Hsu S-T. Standardization work of non-uniform wind loads test on PV module. 37th European Photovoltaic Solar Energy Conference and Exhibition. 2020. pp. 894–899. doi:10.4229/EUPVSEC20202020-4CO.3.2
