# Supplementary material for: Solar cell cracks within a photovoltaic module: Characterization by AC impedance spectroscopy
Source: PLoS One. 2022 Nov 17;17(11):e0277768. doi: 10.1371/journal.pone.0277768 (PMC9671427; doi:10.1371/journal.pone.0277768)
Supplement: S2 Fig — Interconnector ribbons exposed using a micro-grinder (a) and the attached electrical leads (b). (DOCX) [file pone.0277768.s002.docx]

S2 Supporting Information for

“Solar cell cracks within a photovoltaic module: Characterization by AC impedance spectroscopy”

Tadanori Tanahashi, Shu-Tsung Hsu

To electrically separate the individual PV cells within the PV module, the backsheet and encapsulant layers of the PV modules were peeled off (Fig S2a) using a micro-grinder with a small wire brush (Urawa Corp., HD20A-SET), and the electrical leads (1.5 mm in width) were soldered to the interconnector ribbons between the PV cells (Fig S2b). The leads were bound together with another set of copper-solder ribbons (6 mm in width) at each side of the respective PV cells (Fig 1c).


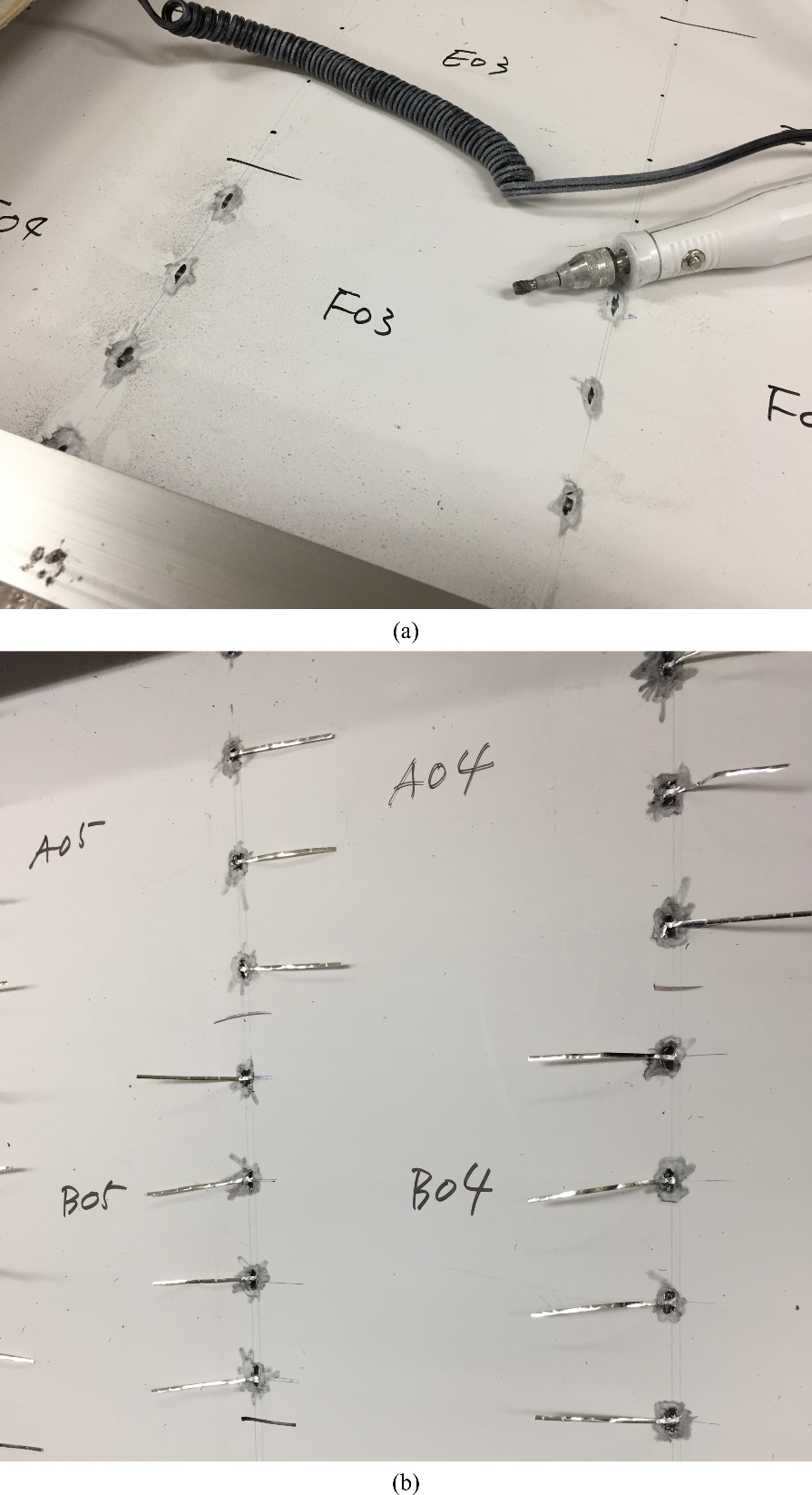


**Fig S2. Interconnector ribbons exposed using a micro-grinder (a) and the attached electrical leads (b).**
